# Multidimensional Factors Associated With Self‐Reported TMD Symptom Burden in Young Adults With DC/TMD‐Defined Myalgia

**DOI:** 10.1111/odi.70404

**Published:** 2026-06-27

**Authors:** Birol Onal, Umit Erkut, Sefa Unes

**Affiliations:** ^1^ Ataturk University Department of Physiotherapy and Rehabilitation Erzurum Türkiye; ^2^ Rumeli University Department of Physiotherapy and Rehabilitation İstanbul Türkiye; ^3^ Bingöl University Faculty of Physical Therapy and Rehabilitation Bingöl Türkiye

**Keywords:** anxiety, masseter muscle, myalgia, neck disability, symptom burden, temporomandibular disorders, ultrasonography

## Abstract

**Background:**

This study aimed to examine multidimensional factors associated with self‐reported Temporomandibular Disorders (TMD) symptom burden, assessed using the Fonseca Anamnestic Index (FAI), in young adults with myalgia defined according to the Diagnostic Criteria (DC) for TMD.

**Methods:**

This cross‐sectional study included 104 young adults with myalgia diagnosed according to the DC/TMD criteria. TMD symptom burden was assessed using the FAI. Clinical, psychosocial, and ultrasonographic variables were evaluated. Backward multiple linear regression was used to identify variables associated with higher FAI scores.

**Results:**

The final model explained 53.0% of the variance in FAI scores (adjusted *R* ^2^ = 0.530, *p* < 0.001). Higher activity‐related pain, greater neck disability, elevated anxiety levels, and a higher number of parafunctional activities were positively associated with higher FAI scores, whereas greater right masseter muscle thickness was inversely associated with FAI scores (*p* < 0.05).

**Conclusions:**

Self‐reported TMD symptom burden among young adults with DC/TMD‐defined myalgia was associated with functional pain, cumulative parafunctional behaviors, neck disability, anxiety, and masseter muscle thickness. These findings support a multidimensional perspective in the assessment of TMD‐related symptoms in individuals with myalgia and highlight the need for longitudinal studies using diagnostically grounded designs.

## 1 Introduction

Temporomandibular disorder (TMD) is a complex clinical condition with a multifactorial etiology that affects both the stomatognathic system and overall health (Palmer and Durham 2021). Affecting a broad segment of the population, this condition may lead to substantial limitations in fundamental oral motor functions, such as mastication, speech, and swallowing, as well as in daily activities and overall quality of life (Bitiniene et al. 2018). Epidemiological evidence indicates that TMD may occur at different stages across the lifespan, often emerging in young adulthood, and is strongly associated with psychosocial factors, including stress, anxiety, and depression, along with lifestyle contributors such as bruxism and postural alterations (Slade et al. 2016; Velly and Fricton 2011).

Pain, joint range of motion, and functional limitations have traditionally constituted the main focus of TMD assessment. However, recent research has highlighted the multidimensional nature of this condition (Peck et al. 2014). Psychological factors, such as anxiety, depression, sleep disturbances, and reduced quality of life, are closely associated with self‐reported TMD symptom burden (Reiter et al. 2015; Sanders et al. 2013). Previous studies have also suggested that TMD‐related symptoms, pain characteristics, and psychosocial profiles may differ according to sex, particularly among young adults (Slade et al. 2016; Yap and Marpaung 2023). In addition, parafunctional habits, including teeth clenching, bruxism, and excessive gum chewing, increase mechanical loading on the temporomandibular joint and masticatory muscles, thereby potentially contributing to TMD‐related symptom expression (Lobbezoo et al. 2018).

Beyond joint involvement, the masticatory muscles play a critical role in the pathophysiology of TMD. The masseter muscle is of particular importance, as it reflects functional loading and parafunctional activity patterns (Manfredini et al. 2011). Therefore, objective assessment of muscle thickness, structural features, and functional alterations is of considerable clinical importance (Castelo et al. 2007). Ultrasonography provides a noninvasive and reliable method for evaluating the morphological and functional features of the masseter muscle (Dong et al. 2015; Kiliaridis and Kälebo 1991). When interpreted alongside clinical findings, these measurements may contribute to a more comprehensive understanding of the mechanisms underlying TMD (Çebi 2019).

Although many studies have examined clinical, pain‐related, and psychosocial factors associated with TMD, fewer studies have evaluated ultrasonographic muscle characteristics alongside these variables within a single multidimensional statistical model (Durham et al. 2016; Slade et al. 2016). While multidimensional approaches to TMD have been previously reported, the combined evaluation of ultrasonographic masseter muscle thickness together with psychosocial, cervical, pain‐related, sleep‐related, and parafunctional factors within a single analytical framework remains uncommon. Multidimensional analytical approaches may therefore enhance understanding of the factors associated with self‐reported TMD symptom burden and support the development of more targeted and comprehensive assessment strategies (Steenks and de Wijer 2009). Accordingly, the present study aimed to examine the independent clinical, psychosocial, and ultrasonographic factors associated with FAI scores in young adults using a comprehensive analytical model.

## 2 Methods

### 2.1 Participants and Study Design

This cross‐sectional descriptive study was conducted among young adults aged 18–40 years. Ethical approval was obtained from the Non‐Interventional Ethics Committee of Atatürk University Faculty of Medicine (B.30.2. ATA.0.01.00/Number: 07/19), and all procedures adhered to the principles of the Declaration of Helsinki. Prior to participation, all individuals were fully informed about the aims and procedures of the study and provided written informed consent.

Participants were recruited from the university and nearby community environments accessible to the research team. Potential participants were informed about the study and screened for eligibility on a voluntary basis. Individuals aged between 18 and 40 years (Arnett et al. 2014; Mehta et al. 2020) with a diagnosis of Diagnostic Criteria for Temporomandibular Disorders (DC/TMD)‐defined myalgia according to the DC/TMD Axis I protocol were included in the study. Only participants presenting with muscle‐related TMD pain were eligible for inclusion. Participants with temporomandibular joint disorders, arthralgia, disc displacement, degenerative joint disease, or other intra‐articular TMD conditions were excluded. Additional exclusion criteria included major psychiatric or neurological disorders, auditory or visual impairments that could interfere with assessment or communication, previous facial or cranial fractures, head trauma, craniofacial surgery, and systemic conditions potentially affecting the musculoskeletal system.

The sample size was determined using G*Power 3.1 software based on an a priori power analysis for multiple linear regression with 13 predictors (Faul et al. 2009). Assuming a medium‐to‐large effect size (*f* ^2^ = 0.25) (Oghli et al. 2025), an alpha level of 0.05, and a statistical power of 0.90, the minimum required sample size was calculated as 102 participants.

### 2.2 Outcome Measures

Demographic and clinical characteristics of the participants, including age, height, body weight, marital status, and mouth opening measurements, were recorded. Mouth opening was assessed in a seated position using a TheraBite Jaw Motion Rehabilitation System (Atos Medical AB, Hörby, Sweden).

TMD diagnosis was established using the DC/TMD Axis I protocol (Schiffman et al. 2014). Only participants diagnosed with DC/TMD myalgia were included in the study. Individuals with temporomandibular joint disorders or other intra‐articular joint problems were not included. All clinical examinations were conducted by a single calibrated clinician (initials) with 14 years of experience in the assessment and management of TMD, who had received formal training in DC/TMD examination procedures prior to data collection. Standardized DC/TMD assessment procedures were applied, including palpation of the masticatory muscles and temporomandibular joint, evaluation of mandibular range of motion, and provocation tests. Based on these assessments, eligibility was confirmed according to the DC/TMD myalgia criteria. In addition to the diagnostic evaluation, self‐reported symptom burden was quantified using the Fonseca Anamnestic Index (FAI). The FAI consists of 10 items scored as 10 (“yes”), 5 (“sometimes”), and 0 (“no”), yielding a total score ranging from 0 to 100, with higher scores indicating greater symptom burden. In the present study, FAI scores were analyzed as a continuous variable to examine gradients in self‐reported TMD‐related symptom burden within a DC/TMD‐defined sample, while acknowledging that the instrument is a screening measure rather than a diagnostic tool (Fonseca et al. 1994).

Parafunctional habits were evaluated using a structured checklist developed specifically for this study. The items were derived from the Oral Behavioral Checklist and adapted for the purposes of the present study (Schiffman et al. 2014). The checklist was based on commonly reported oral parafunctional behaviors described in the literature; however, no formal validation study was conducted. Participants responded to each item in a dichotomous format (“yes” or “no”). Each behavior was coded as present if it was reported to occur at least three times per week. For analysis, the number of behaviors meeting this frequency criterion was summed for each participant to obtain a total parafunctional activity count, representing cumulative parafunctional load. Given the exploratory nature of the study, parafunctional behaviors were operationalized as a cumulative self‐reported activity count rather than as diagnostic entities such as definite or probable bruxism. The internal consistency of the parafunctional behavior checklist was evaluated using Cronbachs alpha and was found to be 0.42.

Masseter muscle thickness was assessed using a B‐mode ultrasonographic device equipped with a high‐frequency linear transducer (Sonosite Edge II; 6–13 MHz). Measurements were obtained with the participant seated in an upright position, in a relaxed jaw posture, with the tongue placed on the hard palate behind the upper teeth and the lips gently closed in a forward‐facing position (An et al. 2021). The transducer was aligned parallel to the line extending from the external auditory meatus to the acanthion and positioned over the masseter region between the zygomatic arch and the mandibular angle. The measurement site was selected at the thickest portion of the masseter muscle, as identified on the ultrasonographic image (Figure 1) (Chang et al. 2020). Muscle thickness was measured three times on both sides, with the order of side assessment randomized, and the mean value was used for analysis (An et al. 2021). Muscle thickness was measured using the built‐in caliper function on frozen ultrasound images obtained during real‐time scanning. All ultrasonographic measurements were performed by a single researcher with 9 years of experience in ultrasonography.

**FIGURE 1:**
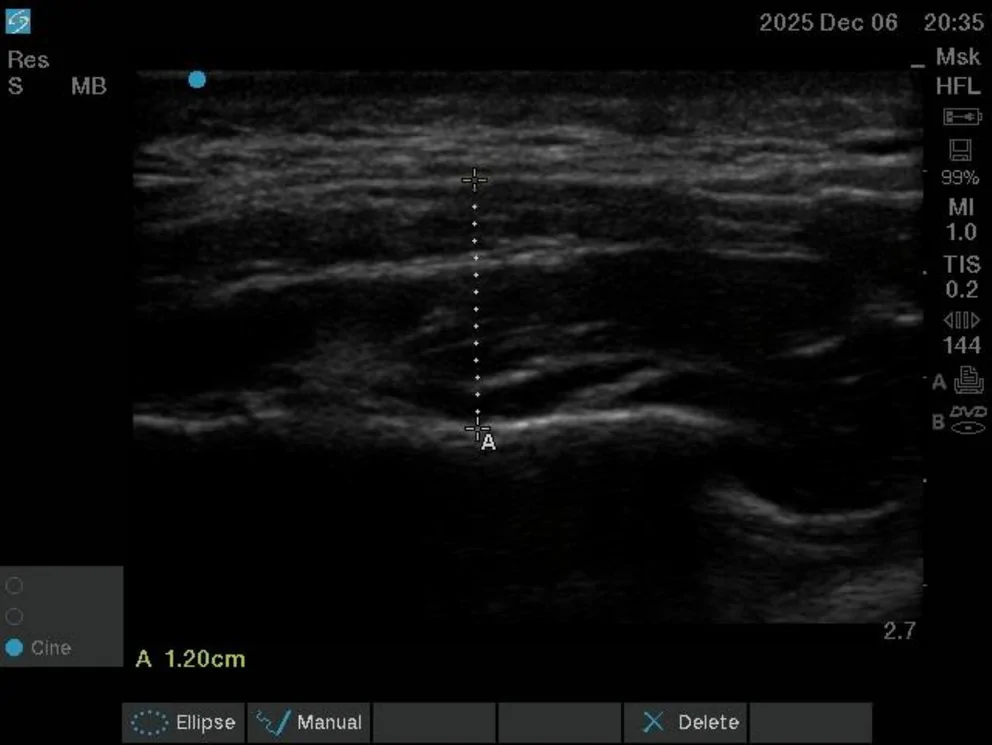
Representative ultrasonographic image of masseter muscle thickness measurement.

Pain intensity was measured with a 100‐mm Visual Analog Scale (VAS), which allowed participants to indicate their pain level on a continuum from no pain (0 cm) to the most severe pain they could imagine (10 cm). Each participant provided separate ratings for pain experienced at rest and during activity (Emshoff et al. 2010).

Neck disability was measured using the Neck Disability Index (NDI), a widely used tool that assesses how neck problems affect daily activities. The instrument asks respondents to rate 10 different aspects of function and symptom severity, such as pain, lifting ability, reading, concentration, driving, and participation in recreational activities. Each item is scored on a 0–5 scale, resulting in a total raw score ranging from 0 to 50, with higher scores indicating greater neck disability (Misterska et al. 2011).

Anxiety and depression levels were assessed using the Hospital Anxiety and Depression Scale (HADS), which consists of 14 items. Seven of these items measure anxiety (HADS‐A), and the remaining seven measure depression (HADS‐D). Each subscale yields a score ranging from 0 to 21, and higher scores indicate more severe symptoms (Zigmond and Snaith 1983).

Sleep quality and insomnia symptoms were evaluated using the Athens Insomnia Scale (AIS), a self‐reported measure based on ICD‐10 criteria. The scale comprises eight items in total, including five related to difficulties experienced during sleep and three that assess daytime consequences. Each item is rated on a scale from 0 to 3, producing a total score ranging from 0 to 24. A score of 6 or above is generally considered indicative of an increased risk of insomnia (Soldatos et al. 2003).

### 2.3 Statistical Analysis

All statistical analyses were performed using IBM SPSS Statistics (version 26.0). Continuous variables were summarized as mean ± standard deviation, and categorical variables as frequencies and percentages. Data normality was assessed using the Shapiro–Wilk test and visual inspection of histograms and Q–Q plots. Pearson correlation coefficients were calculated to examine associations between FAI scores and the clinical and psychosocial variables. Comparisons between sexes were conducted using independent samples *t*‐tests. Categorical variables, including the distribution of individual parafunctional habits, were compared using the chi‐square test.

Factors independently associated with FAI scores were explored using backward multiple linear regression. The initial model included age, body mass index, sex, number of parafunctional activities, right masseter muscle thickness, left masseter muscle thickness, mouth opening, VAS pain at rest, VAS pain during activity, neck disability (NDI), HADS‐depression score, HADS‐anxiety score, and insomnia severity (AIS). The FAI total score was entered as the dependent variable to represent self‐reported symptom burden. A *p*‐out value of 0.10 was applied to remove noncontributing variables. Regression assumptions were checked, including multicollinearity, normality of residuals, homoscedasticity, and autocorrelation. No problematic multicollinearity was observed in the final model, with all tolerance values > 0.70 and VIF values < 1.5. Given the exploratory aim of the study, the regression model was interpreted as identifying variables associated with higher FAI scores, rather than establishing causal or diagnostic relationships. A significance level of *p* < 0.05 was used for all analyses.

## 3 Results

A total of 160 individuals were assessed for eligibility. Of these, 28 were excluded for not meeting the eligibility criteria, 18 declined participation, and 10 were not included because of incomplete assessment data or logistical reasons. Consequently, a total of 104 young adults who met the DC/TMD criteria for myalgia participated in the study, with a mean age of 25.7 ± 6.5 years. The sample included equal numbers of females and males. The demographic, clinical, and psychosocial characteristics of the study sample are summarized in Table 1.

**TABLE 1:** Demographic, clinical, and psychosocial characteristics of the study sample.

	Mean (SD)
Age (years)	25.7 (6.5)
Height (cm)	170.6 (9.5)
Weight (kg)	69.7 (4.9)
Body mass index (kg/m^2^)	23.8 (3.7)
Sex	*n* (%)
Female	52 (50.0)
Male	52 (50.0)
Marital status	
Married	15 (14.4)
Single	89 (85.6)
	**Mean (SD)**
FAI score (0–100 points)	28.7 (20.5)
Number of parafunctional activities (0–8)	1.9 (1.4)
Mouth opening (mm)	48.8 (6.8)
VAS at rest (0–10 points)	0.4 (1.1)
VAS during activity (0–10 points)	1.0 (2.0)
NDI score (0–50 points)	6.8 (5.3)
HADS‐depression (0–21 points)	6.3 (3.1)
HADS‐anxiety (0–21 points)	7.1 (3.7)
AIS (0–24 points)	5.8 (3.7)

Abbreviations: AIS, athens insomnia scale; FAI, fonseca anamnestic index; HADS, Hospital Anxiety and Depression Scale; NDI, Neck Disability Index; VAS, Visual Analog Scale.

Sex‐based comparisons of clinical and psychosocial variables are presented in Table 2. Compared with men, women showed higher FAI scores, reduced mouth opening, higher neck disability scores, and higher anxiety levels, whereas men demonstrated higher depression scores. No significant sex differences were identified in parafunctional activity count, pain intensity, or insomnia severity. The distribution of individual parafunctional habits was also comparable between sexes, with cheek, lip, or tongue biting being the most commonly reported habit among women and gum chewing among men (Table 3).

**TABLE 2:** Sex‐based comparison of clinical and psychosocial characteristics.

	Women mean (SD)	Men mean (SD)	*t*	*p*	*d*
FAI score (0–100 points)	32.79 (23.08)	24.62 (16.68)	2.07	**0.041**	0.41
Number of parafunctional activities (0–8)	1.88 (1.31)	1.84 (1.55)	0.15	0.884	0.03
Mouth opening (mm)	47.23 (7.64)	50.40 (5.50)	−2.40	**0.018**	0.46
VAS at rest (0–10 points)	0.44 (1.18)	0.33 (1.05)	0.50	0.622	0.10
VAS during activity (0–10 points)	1.13 (2.22)	0.76 (1.83)	0.92	0.359	0.18
NDI score (0–50 points)	7.90 (5.62)	5.67 (4.66)	2.20	**0.030**	0.44
HADS‐depression (0–21 points)	5.35 (2.60)	7.29 (3.18)	−3.40	**0.001**	0.67
HADS‐anxiety (0–21 points)	7.94 (3.52)	6.18 (3.77)	2.46	**0.016**	0.48
AIS (0–24 points)	5.50 (3.27)	6.20 (4.08)	−0.96	0.341	0.19

*Note:* Independent samples *t*‐tests were used. Cohen's d: effect size (0.2 small, 0.5 medium, 0.8 large). Statistically significant *p* values are shown in bold.

Abbreviations: AIS, athens insomnia scale; FAI, fonseca anamnestic index; HADS, Hospital Anxiety and Depression Scale; NDI, Neck Disability Index; VAS, Visual Analog Scale.

**TABLE 3:** Sex‐based comparison of parafunctional activities.

Parafunctional activity	Total *n* (%)	Women *n* (%)	Men *n* (%)	*p*
Teeth clenching	34 (32.7)	19 (36.5)	15 (28.8)	0.403
Teeth grinding	7 (6.7)	2 (3.8)	5 (9.6)	0.240
Gum chewing	41 (39.4)	19 (36.5)	22 (42.3)	0.547
Unilateral chewing	34 (32.7)	17 (32.7)	17 (32.7)	1.000
Cheek/lip/tongue biting	40 (38.5)	23 (44.2)	17 (32.7)	0.227
Biting pens or other objects	10 (9.6)	6 (11.5)	4 (7.7)	0.506
Nail biting	11 (10.5)	3 (5.8)	8 (15.4)	0.111
Frequent consumption of hard foods	15 (14.4)	9 (17.3)	6 (11.5)	0.402

*Note:* Chi‐square tests were used.

FAI scores were positively correlated with the number of parafunctional activities (*r* = 0.37, *p* < 0.001), pain intensity at rest (*r* = 0.42, *p* < 0.001), pain intensity during activity (*r* = 0.56, *p* < 0.001), NDI (*r* = 0.55, *p* < 0.001), HADS‐A (*r* = 0.33, *p* = 0.001), and AIS (*r* = 0.27, *p* = 0.006). It was negatively correlated with mouth opening (*r* = −0.29, *p* = 0.003) and masseter muscle thickness on both sides (right: *r* = −0.32, *p* = 0.001; left: *r* = −0.23, *p* = 0.020) (Table 4). No significant correlation was found between FAI scores and depression.

**TABLE 4:** Correlations between FAI scores and clinical or psychosocial variables.

Variables	*r*	*p*
Age	0.09	0.34
BMI	0.01	0.91
Number of parafunctional activities	0.37	**< 0.001**
Mouth opening	−0.29	**0.003**
Right masseter thickness	−0.32	**0.001**
Left masseter thickness	−0.23	**0.018**
VAS at rest	0.42	**< 0.001**
VAS during activity	0.56	**< 0.001**
NDI score	0.55	**< 0.001**
HADS‐depression	0.15	0.12
HADS‐anxiety	0.33	**0.001**
AIS	0.27	**0.006**

*Note:* Statistically significant *p* values are shown in bold.

Abbreviations: AIS, athens insomnia scale; BMI, body mass index; HADS, Hospital Anxiety and Depression Scale; NDI, Neck Disability Index; *r*, pearson correlation coefficient; VAS, Visual Analog Scale.

The final regression model significantly explained 53.0% of the variance in FAI scores (adjusted *R* ^2^ = 0.530, *F* (5, 98) = 24.35, *p* < 0.001) (Table 5). Higher parafunctional activity count (*β* = 0.151, *p* = 0.040), greater pain during activity (*β* = 0.424, *p* < 0.001), higher neck disability (*β* = 0.267, *p* = 0.002), and higher anxiety scores (*β* = 0.161, *p* = 0.038) were independently associated with higher FAI scores. In contrast, right masseter muscle thickness was inversely associated with FAI scores (*β* = −0.162, *p* = 0.027).

**TABLE 5:** Multiple linear regression model for factors associated with FAI scores.

Predictor	*B*	SE *B*	β	*t*	*p*	95% CI	Tolerance	VIF
Constant	32.481	12.910		2.516	0.014			
Number of parafunctional activities	2.304	1.107	0.151	2.081	0.040	0.105–4.503	0.907	1.103
USG right masseter thickness	−27.937	12.452	−0.162	−2.244	0.027	−52.660 – −3.214	0.905	1.105
VAS during activity	4.241	0.761	0.424	5.573	< 0.001	2.730–5.752	0.820	1.219
NDI	1.036	0.317	0.267	3.263	0.002	0.406–1.666	0.707	1.415
HADS‐anxiety	0.897	0.426	0.161	2.108	0.038	0.052–1.742	0.809	1.236
Model statistics: *R* = 0.744, *R* ^2^ = 0.554, adjusted *R* ^2^ = 0.530, *F*(5, 98) = 24.35, *p* < 0.001								

*Note:* Tolerance values were > 0.20 and all VIF values were < 5, indicating no problematic multicollinearity among the independent variables.

Abbreviations: B, unstandardized regression coefficient; *β*, standardized regression coefficient; CI, confidence interval; HADS, Hospital Anxiety and Depression Scale; NDI, Neck Disability Index; SE B, standard error of B; USG, ultrasonography; VAS, Visual Analog Scale; VIF, variance inflation factor.

## 4 Discussion

TMD are increasingly recognized as multifactorial conditions arising from the interaction of biomechanical, behavioral, and psychosocial factors. In young adults, TMD‐related complaints may often be reflected more prominently in self‐reported symptom measures than in clearly differentiated clinical subgroups. In the present study, activity‐related pain and cumulative parafunctional loading were prominently associated with higher FAI scores, with additional contributions from anxiety, neck disability, and masseter muscle thickness. Together, these findings highlight the importance of a multidimensional perspective in understanding TMD‐related symptom expression, in which functional, musculoskeletal, and psychosocial factors may interact within a diagnostically confirmed myalgia sample with heterogeneous symptom burden.

The relatively low pain intensity scores observed in the present study may reflect the characteristics of the study sample. Unlike patients recruited from specialized TMD clinics, the participants in this study were community‐dwelling young adults identified through DC/TMD‐based assessment and were not necessarily seeking treatment for TMD‐related complaints. Previous studies conducted in clinical TMD populations have generally reported substantially higher pain intensity levels, particularly among chronic and treatment‐seeking patients (Ferrillo et al. 2026; Lee et al. 2025). Therefore, the symptom profile of the present sample may represent a relatively milder clinical presentation despite meeting the diagnostic criteria for myalgia. Nevertheless, activity‐related pain emerged as the variable most strongly associated with self‐reported TMD symptom burden in this cohort, consistent with previous research (Yap, Tan, and Marpaung 2022; Yap, Xiong, et al. 2025). Functional pain during activities such as mastication and speech has been shown to contribute more substantially to symptom burden and functional limitation than resting pain, particularly in young adults (Yap, Lei, et al. 2025; Yap, Tan, and Marpaung 2022; Yap, Xiong, et al. 2025). Similar findings have been reported across different TMD presentations in the literature, suggesting that activity‐related pain may be a common correlate of greater symptom burden (Schiffman et al. 2014; Yap, Zhang, et al. 2022). Increased mechanical loading of the masticatory muscles during functional activity may heighten nociceptive sensitivity, which may help explain the prominent association of activity‐related pain with higher FAI scores observed in this study.

An important finding of the present study was the inverse association between right masseter muscle thickness and FAI scores. However, this finding should be interpreted within the complex clinical context of myogenous TMD. Previous ultrasonographic studies have reported heterogeneous findings regarding masseter muscle morphology in individuals with TMD, with some studies demonstrating no significant differences in muscle thickness between myalgia patients and healthy controls or between unilateral and bilateral TMD presentations (Arıkan et al. 2023; Fathy et al. 2025). These inconsistencies suggest that ultrasonographic muscle characteristics may be influenced by multiple interacting factors, including symptom chronicity, parafunctional loading, pain‐related functional adaptation, muscle stiffness, edema, and bruxism‐related mechanical loading (Imanimoghaddam et al. 2013; Olchowy et al. 2020). In some cases, parafunctional loading may contribute to muscle hypertrophy, whereas chronic pain‐related disuse or adaptive jaw‐use behavior may be associated with reduced muscle thickness (Çebi 2019; Ohrbach et al. 2011). Furthermore, ultrasonographic findings in TMD may also be influenced by psychosocial factors such as anxiety, which can contribute to altered muscle activity, pain‐related functional inhibition, and variable muscular adaptation patterns (Öçal et al. 2025).

In the present study, cumulative parafunctional activity count was included in the regression model; however, symptom duration, pain chronicity, myalgia subtype, number of painful palpation sites, pain localization, and side‐specific symptom characteristics were not systematically recorded. Therefore, the observed inverse association should not be interpreted as a direct or causal relationship between reduced masseter thickness and symptom severity alone, but rather as a potential association with multidimensional self‐reported TMD symptom burden within a DC/TMD‐defined myalgia sample.

The fact that only the right masseter thickness remained significant in the final regression model also warrants cautious interpretation. This finding does not necessarily indicate a consistent right‐sided pathological mechanism. Rather, it may reflect asymmetric functional loading patterns, individual chewing‐side preference, side‐specific parafunctional activity, or pain‐related adaptive jaw‐use behavior. In the present study, unilateral chewing habits were reported by approximately one‐third of participants in both sexes, suggesting that asymmetric functional loading may be relatively common in this population. However, chewing‐side dominance, side‐specific pain localization, and detailed parafunctional laterality patterns were not assessed. Previous ultrasonographic studies investigating masseter morphology in myogenous TMD have similarly reported asymmetric and inconsistent findings. Heterogeneous laterality patterns in masseter width measurements have been reported, with side‐dependent differences not consistently reproduced across contraction conditions (Poveda‐Roda et al. 2018). Significantly reduced right‐sided masseter thickness has been reported in females with myofascial pain disorder, whereas the contralateral side did not demonstrate comparable significance (Imanimoghaddam et al. 2013). Collectively, these findings suggest that ultrasonographic alterations in masseter morphology may not occur uniformly across both sides and may instead reflect functional laterality, chewing dominance, interindividual variability, and pain‐related adaptive responses rather than a consistent bilateral pathological process. Therefore, the lack of statistical significance for the left masseter muscle should not necessarily be interpreted as evidence of absence of involvement, but may instead reflect the inherently heterogeneous and asymmetric nature of muscular adaptation in myogenous TMD (De Nordenflycht et al. 2024; Lee et al. 2019). Potential multicollinearity between right and left masseter muscle thickness variables was assessed during regression diagnostics using tolerance and variance inflation factor (VIF) values, and no problematic multicollinearity was identified. Therefore, the unilateral association observed in the present study is unlikely to be attributable solely to statistical collinearity, although confirmation in larger studies incorporating detailed side‐specific clinical and functional assessments remains necessary. In addition, a relatively large number of statistical tests were performed in the present study. Therefore, the possibility that the observed association between right masseter muscle thickness and FAI scores may partially reflect experiment‐wise type I error cannot be completely excluded and should be considered when interpreting this finding.

The total number of parafunctional activities was independently associated with higher FAI scores, supporting the concept of cumulative behavioral loading rather than the isolated effect of single parafunctions such as bruxism alone (Boening et al. 2015; Zemowski et al. 2025). Previous studies have suggested that the coexistence of multiple parafunctional behaviors may contribute to repetitive microloading and increased muscular demand, thereby increasing symptom burden (Zemowski et al. 2025). From this perspective, behaviors such as gum chewing, unilateral chewing, nail biting, and object biting may be more relevant in combination than when considered individually (Zemowski et al. 2025; Zheng et al. 2023). In addition, sustained oral parafunctional loading may increase mechanical stress on the masticatory system and contribute to pain sensitivity (Zieliński et al. 2024). Collectively, these findings suggest that the cumulative burden of parafunctional behaviors may be important in understanding variability in self‐reported TMD symptom burden.

Anxiety also emerged as an independent factor associated with higher FAI scores, consistent with extensive evidence linking TMD‐related symptoms with anxiety and emotional distress in young adult populations (Mortimer‐Jones et al. 2019; Paludo et al. 2024; Pesqueira et al. 2010; Yap and Marpaung 2023). Moreover, anxiety may be associated with symptom burden not only through heightened pain perception but also through behavioral pathways such as increased parafunctional activity, including bruxism‐related behaviors (Hoa et al. 2025). In addition, previous research suggests that the relationship between TMD and anxiety may be bidirectional, with TMD‐related symptoms and anxiety potentially reinforcing one another over time (Liou et al. 2023). Notably, prospective, longitudinal, and comparative evidence suggests a temporal differentiation in the psychosocial profile of TMD. Anxiety appears to be more strongly associated with the development and early manifestations of TMD symptoms, whereas depressive symptoms may become increasingly prominent as pain persists and progresses toward a more chronic condition (Cao et al. 2021; de Lucena et al. 2012; Kindler et al. 2012; Velly et al. 2011). This pattern may help explain why anxiety, but not depression, emerged as an independent predictor in the present study, particularly given the relatively young age and community‐based characteristics of the sample. Together, these findings suggest that anxiety may play a more prominent role than depression in the expression of self‐reported TMD symptoms among young adults.

Neck disability emerged as an independent factor associated with higher FAI scores in the present study, consistent with evidence supporting the functional and biomechanical interrelationship between the jaw and cervical systems. Altunalan et al. (2025) reported that individuals with greater neck disability were more likely to exhibit TMD‐related symptoms, with higher NDI scores associated with greater symptom burden. Similarly, Olivo et al. (2010) demonstrated a strong association between neck disability and jaw‐related dysfunction, highlighting the close functional interaction between the cervical and mandibular regions. Kaynak et al. (2024) identified alterations in cervical muscle morphology and increased muscular loading in individuals with TMD, suggesting that cervical dysfunction may contribute to TMD‐related symptom expression. Together, these findings reinforce the importance of considering the cervicocraniomandibular region in the multidimensional assessment of TMD‐related symptoms, particularly in young adults.

### 4.1 Limitations

This study has several limitations. First, parafunctional behaviors were assessed using a self‐reported checklist, which may not fully capture subconscious or habitual activities such as sleep bruxism. In addition, the checklist was developed for the purposes of this study, and no formal validation study was performed. Therefore, the cumulative parafunctional activity score should be considered a pragmatic self‐reported indicator of behavioral loading. Second, the ultrasonographic assessment was limited to static masseter muscle thickness and did not include functional or mechanical properties such as muscle activity, stiffness, or contractile behavior. In addition, temporalis and other masticatory muscles were not evaluated ultrasonographically in the present study. Future studies incorporating multiple masticatory muscles and more comprehensive ultrasonographic approaches may provide a broader understanding of muscle‐related characteristics associated with TMD symptom burden. Furthermore, although all DC/TMD and ultrasonographic assessments were performed by single experienced examiners to ensure consistency, formal intra‐rater reliability analyses and reassessment procedures to monitor potential examiner drift were not conducted during the study period. This methodological limitation is recognized, and the results should be interpreted with caution regarding examiner‐related variability. Third, although all participants met the DC/TMD criteria for myalgia, more detailed myalgia‐specific clinical characteristics, such as symptom duration, pain chronicity, and myalgia subtypes, were not systematically recorded. Therefore, the clinical characterization of the myalgia sample remained limited. Finally, although TMD diagnosis was established using the DC/TMD, self‐reported symptom burden was quantified using the FAI, which is a symptom‐based screening instrument rather than a diagnostic severity tool. Accordingly, the findings should be interpreted as reflecting associations with self‐reported symptom burden within a DC/TMD‐defined myalgia sample.

## 5 Conclusion

This study showed that self‐reported TMD symptom burden in young adults is associated with a combination of activity‐related pain, cumulative parafunctional activity load, neck disability, anxiety, and masseter muscle thickness. Among these factors, activity‐related pain, behavioral loading, and neck disability were prominently associated with higher FAI scores in this population. The observed inverse association between right masseter muscle thickness and self‐reported symptom burden suggests that ultrasonographic assessment may contribute to a better understanding of musculoskeletal features associated with TMD‐related symptoms, although its clinical implications require further investigation. Overall, these findings support a multidimensional assessment perspective integrating clinical, behavioral, musculoskeletal, and psychosocial factors. Future longitudinal studies incorporating advanced imaging techniques and diagnostically grounded designs are needed to further clarify these associations.

## Author Contributions

**Sefa Unes:** conceptualization, data curation, formal analysis, investigation, project administration, supervision, visualization, writing – original draft, writing – review and editing. **Umit Erkut:** conceptualization, investigation, methodology, resources, writing – review and editing. **Birol Onal:** conceptualization, data curation, formal analysis, investigation, methodology, visualization, writing – original draft, writing – review and editing.

## Funding

The authors received no financial support for the research, authorship, and/or publication of this article.

## Ethics Statement

The study was approved by the Non‐Interventional Clinical Research Ethics Committee of Atatürk University Faculty of Medicine (approval no. B.30.2.ATA.0.01.00/07/19). The study was conducted in accordance with the principles of the Declaration of Helsinki.

## Consent

Written informed consent was obtained from all participants prior to participation in the study.

## Conflicts of Interest

The authors declare no conflicts of interest.

## Supporting information


**Data S1:** STROBE Statement—checklist of items that should be included in reports of observational studies.

## Data Availability

The data that support the findings of this study are available from the corresponding author upon reasonable request.
